# A Novel Methodology for Measuring the Abstraction Capabilities of Image Recognition Algorithms

**DOI:** 10.3390/jimaging7080152

**Published:** 2021-08-19

**Authors:** Márton Gyula Hudáky, Péter Lehotay-Kéry, Attila Kiss

**Affiliations:** 1 Department of Information Systems, ELTE Eötvös Loránd University, 1117 Budapest, Hungary; bfspl6@inf.elte.hu (M.G.H.); lkp@student.elte.hu (P.L.-K.); 2 Department of Informatics, J. Selye University, 94501 Komárno, Slovakia

**Keywords:** artificial intelligence, neural networks, abstraction, image recognition

## Abstract

Creating a widely excepted model on the measure of intelligence became inevitable due to the existence of an abundance of different intelligent systems. Measuring intelligence would provide feedback for the developers and ultimately lead us to create better artificial systems. In the present paper, we show a solution where learning as a process is examined, aiming to detect pre-written solutions and separate them from the knowledge acquired by the system. In our approach, we examine image recognition software by executing different transformations on objects and detect if the software was resilient to it. A system with the required intelligence is supposed to become resilient to the transformation after experiencing it several times. The method is successfully tested on a simple neural network, which is not able to learn most of the transformations examined. The method can be applied to any image recognition software to test its abstraction capabilities.

## 1. Introduction

The development of artificial intelligence systems can be traced back to the 1940s. The early stages of development were dominated by the creation of independent, unchanging, and task-specific programs. In this approach, learning can be interpreted as a pure memorization task according to early pioneer Marvin Minsky [1].

The first famous achievement of the field of artificial intelligence was the famous ELIZA (named after Eliza Doolittle, a working-class character in George Bernard Shaw’s Pygmalion) program developed by Weizenbaum et al. in the 1960s, which can process the English language and mingle in conversation with another program or even with a human [2]. Another big success was the Deep Blue chess program, which was able to beat the world champion, Garry Kasparov, in 1997 [3,4]. However, the intelligence of these programs is highly questionable, as they are only able to solve certain tasks the programmer prepared them for.

After the turn of the millennium, researchers in artificial intelligence increasingly began to strive to create more general systems with better abstraction capabilities. A good example of this endeavor is the Alpha Zero program. Silver et al. tested it on chess, shogi, and go, but others thought the algorithm many more games since then [5,6]. Alpha Zero is a more general intelligence than Deep Blue was since it can understand more domains.

Artificial intelligence systems have developed rapidly in recent decades and the need of creating tests to measure intelligence became more and more relevant. However, the definition of intelligence is already a difficult task, many interpretations exist from different viewpoints and sciences [7]. In the present paper, we adhere to the definition of intelligence as the ability to convert experience into knowledge. We view knowledge as an understanding of a system, that is, in possession of knowledge, we can correctly predict the behavior of the system. By comparing the responses given by a system and the responses predicted by the intelligent actor, we can therefore examine whether the intelligent actor understood the system. Furthermore, if we examine what resources the system needs to acquire this knowledge, we can deduce the intelligence of the system.

The concept of abstraction is closely related to intelligence and expresses the extent to which an actor can generalize what he has learned in a given task and apply it to other types of tasks [8]. We are also able to classify intelligent actors according to their abstraction capabilities. Solutions without any abstraction can perform excellently on a single task if either the programmer has to code the rule system into the intelligent actor, or the system can calculate all the possibilities using a large number of resources. In contrast, general intelligence systems are capable of human-like abstraction and can achieve good results in a whole variety of tasks without having to analyze the event space in depth.

The development of tests measuring intelligence and abstraction was not one of the primary objectives of the field. The creation of such tests would greatly help us to understand how artificial intelligence systems work and to be able to create more general artificial intelligence. Thus, a system may be able to replace a system developed for several specific tasks, or it may be able to solve many tasks that we did not know before.

Our goal was to create a system in which we can effectively measure the abstraction capability of different software. By abstraction capability, we mean the ability of software to apply learned rules in different situations. Previous tests of a similar nature have been based on how much knowledge the software has been able to acquire in a given environment. A problem with this approach is that learning can be avoided if the programmer pre-encapsulates the knowledge into the software. Our approach aims to filter out these pre-written solutions. We did so by measuring the performance of the same network under different circumstances and calculating how much progress the system is able to make with extra information. This way pre-written solutions will fail the test since they perform well with little information and do not make further progress with additional information.

## 2. Related Works

### 2.1. Task-Specific Tests

The early approaches of measuring intelligence were task-specific tests since the systems tested were also single-task purpose programs [9].

One of the first attempts to define artificial intelligence is attributed to Alan Turing, a test originally known as a “simulation game,” known as the Turing test [10]. The essence of Turing and similar tests is that an intelligent system behaves in such a way that it cannot be distinguished from human behavior by a human observer. There have been several variations of the Turing test and the Loebner Award, which is awarded to the chatbot that performs best on the Turing test each year [11]. In fact, human behavior must be sufficiently mimicked by the system. However, these tests circumvent the problem of defining intelligence and leave it to an external subjective factor: human judges. Already in the early stages of the development of artificial intelligence, some programs managed to pass the Turing test without applying any abstraction to it.

Another common approach is to compete with intelligent systems. This is typically solved in the case of games using threads played against each other. For example, they measure the knowledge of chess programs in this way, but many other competitive games also have advanced intelligence systems that can play against each other or people. Moreover, the Alpha Go Zero chess machine has appreciated various move options and acquired superhuman abilities during the games against itself. The primary problem with this evaluation method is that the result can only be evaluated with the knowledge of the opponent, but it is still difficult to quantify. In chess and many other games, the ELO (named after its creator Arpad Elo) system is used, so if the same players have played several games with each other, the system assigns a score to them, and we can also determine the order of the players based on their scores. The biggest disadvantage of this method is that players will be made specifically against each other, as is the case with human chess players. In the case of programs, this can often mean specializing in old, poorly approached programs [9].

The most common task-specific approach is problem-solving measurements. Here, we define the tasks and their solutions in advance, and then we examine which tasks the system was able to solve correctly. Although this method eliminates the dependence of evaluation on people or other intelligent systems, as it depends only on the objective solution, the dependence on the task itself will be very strong. For example, there may be faulty task solutions, tasks available in public places that can be pre-programmed into the system, or an event space that can be traversed by an exhaustive search. Here, too, the possibility of a “big shift” solution method arises, in which case, the program only recognizes which type of task it is and then uses a pre-programmed solution to solve the given task. With this, the system can look intelligent, as the task can be arbitrarily different. Task-solving measurements work best when the selection of test cases and the setting up of the intelligence system are done by the same team, as there is an extremely high chance that a specialized solution will be created for the tasks [9,12].

### 2.2. Ability-Based Tests

The first experiments to measure the cognitive abilities of an artificial intelligence system rather than task-specific abilities are rooted in human psychometry and are similar to human IQ tests. These IQ tests do work well in a timeless way to measure the intelligence of human subjects but are not particularly useful for artificial intelligence systems. Sanghi and Dowe created a system in 2003 that uses the “big shift” technique, which has performed well on various IQ tests but is not considered intelligent by the authors. However, IQ tests also have the disadvantage that they take a lot of information for granted in the context; for example, if they contain a text part, they take knowledge of the language of the text as given. An important consideration in setting up an environment for testing artificial intelligent systems is what kind of information we take for granted. The fewer such assumptions are given, the more generally the test will be applicable. However, knowledge of a human language is a complex task in itself, so it will not be part of a test measuring artificial intelligence [13].

A whole new approach was born in the early 1990s. The algorithmic information theory sees intelligence as a process of information processing. There are several definitions of information and complexity that can be traced back to computational tasks; typically, computational tasks are performed by a universal Turing machine. Thus, for example, the complexity of a task is proportional to the shortest program that solves the task. Of course, these complexity values may be different for different Turing machines, but they differ only by a constant in the sense of the invariance theorem. The complexity values measured in this way correlate well with how difficult the task proved to be for humans [9,14].

It was not until the 2000s that efforts began to create universal psychometric tests that could be used to measure both human and artificial intelligence, or, as Hernández-Orallo and Dowe stated, applicable to any biological or artificial system that exists now or in the future. To this end, any cultural, linguistic, or species-specific task must be excluded. Furthermore, testing can only be solved effectively using an adaptive method, as subjects can have extremely different intelligence, so the difficulty of the tasks during testing must be adjusted to the subject’s abilities [15]. The more we know about the subject being tested, the easier is it to test its intelligence. That is why we can write good intelligence tests on humans. Similarly, certain tests are able to measure subjects well in a specific, well-known class of artificial intelligence. Creating a test that can test and measure the intelligence of extremely different subjects is very challenging.

According to an interesting computer theory, the No Free Lunch theorem by David Wolpert, if an algorithm performs better in solving a group of tasks, it necessarily involves performing worse on other tasks. The theory became popular and frequently referred to in the 1990s, but its validity and scope have been debated ever since. Under certain conditions, there may be “Free Lunches”, that is, situations where an algorithm performs significantly better than other algorithms on a task group, such as certain coevolution algorithms or algorithms developed with supervised learning. The main message of the No Free Lunch theory is that effectiveness is determined by specific problems; for example, human effectiveness can only be interpreted in the space of tasks known to man [16,17,18].

One of the most recent studies about measuring intelligence is the Abstraction and Reasoning Challenge (ARC). Chollet et al. created tasks consisting of three example image pairs and a test input image. Each example image pair consists of two images, one representing the input and the other the output. From the three examples, the system needs to figure out what the logic might be, based on which the output can be made. We are doing some transformation on the input image, and this transformation needs to be understood by the system. Whether you have understood the transformation is to be checked by the test example. Here, only the input image is given, the system has to guess the output [19]. A Kaggle Challenge was initiated where developers tried to crack ARC tasks with intelligent systems. Unfortunately, solutions for ARC tasks typically do not bring us closer to inventing general artificial intelligence. They use some kind of search algorithm, where the developer defines the possible transformations, and the program tries them out to find the right combination of transformations to solve the task.

Another powerful tool to crack ability-based tasks without truly learning the ability is the “big switch” method. It can be applied when the tasks can be categorized into well-defined categories. In the “big switch” system the main program keeps track of which subprogram can solve which task and, by identifying the type of task, solves it with the appropriate subprogram and reaches the correct conclusion. The “big shift” method increased the applicability of artificially intelligent systems but did not bring it closer to general artificial intelligence, as the program was still only able to solve those tasks whose solution was written by the programmer [9].

### 2.3. Hard-Coded Solutions

Several hard-coded solutions exist for recognizing transformations of an object. However, these are pre-written by the developer so the intelligent system is only using these methods.

One hard-coded solution is the use of fuzzy systems, created by combining supervised and unsupervised learning [20]. Patil et al. developed invariant solutions for rotation, movement, and resizing. Marcos et al. made a conventional neural network invariant for rotation with a specific inner structure [21]. Worrall et al. reached rotational invariance by requiring the neural network layers to be symmetric. This will require a slightly higher computational requirement, but it is assured that we get the same results at a full 360° [22].

A very interesting and much-researched feature of image recognition neural networks is that they can be achieved with minimal but well-targeted changes to miscategorize images. That is, if we know the structure of the neural network, we can add small differences to an image depicting a certain shape (which the neural network correctly classifies) that the human observer does not even notice, but this is enough to deceive the neural network and categorize the image incorrectly. Networks robust to such attacks are being investigated, including in the case of the MNIST (Modified National Institute of Standards and Technology). database [23,24] which is a large database containing handwritten digits used for training and testing image processing systems, also in the field of machine learning.

Face recognition is one of the most researched applications of image recognition and one of the most active research fields of computer vision [25]. Practical applications include identification, access control, and human–computer interactions. In one of the most recent studies, Zhang et al. target recognition of microexpression on the human face [26].

Cancer prognosis is another very impactful research area of image recognition. Multiple types of cancer can be recognized with the help of image recognition [27,28,29], but there are also other AI-based applications, such as pulse signal processing for the recognition of lung cancer [30].

In the field of additive manufacturing, one of the challenges is properly detecting errors. In 3D printing, there is a chance of creating defected elements that have to be detected and replaced with properly created elements. Straub et al. examined the detection of defected elements with image analysis [31].

## 3. Concepts

The experiments detailed in the literature review to measure intelligence did not live up to their expectations. Typically, the puzzles proved to be solvable with traditional methods without creative artificial intelligence, as shown in the case of ARC. A fundamental problem is that we can only invent and evaluate tasks that can be solved by humans, but then one can code one’s solution mechanism. This can be partially resolved if the tasks are very different from each other and the programmer does not know them in advance, as in this case, he does not think about certain types of tasks and thus does not code them into the program.

Another approach could be to consider from the outset not the result achieved by the intelligent software on a specific task, but the result is the development of its performance. Intelligence was defined as the efficient processing and conversion of information into knowledge. In previous tests, this could be circumvented by not having or only partially needing to process the information if the system had some knowledge in advance. Therefore, it may be appropriate not to measure ultimate knowledge but to measure knowledge growth, as ultimately, this is what is proportional to intelligence.

One solution to measure the increase in knowledge may be to measure the knowledge of the system at a certain point, continue to teach the system, and then measure its knowledge again. This solution can give very accurate results; however, if the intelligent system developer is well acquainted with the test environment, it can be circumvented with software that intentionally gives weaker answers for the first time.

Another solution for measuring knowledge growth is to teach two separate instances of the system with smaller or larger amounts of information, respectively. Then there may be minor differences between the two specimens if their operation is non-deterministic, but we avoid the playability of the test system, which is our main goal in our research. Individual specimens are unaware that they will receive a higher value on the intelligence test for good or bad responses because they do not know whether they are the lower or upper reference point of the measurement.

Of course, this type of measurement raises a lot of questions, such as how to compare different performance gains measured from different starting points or what we can do with systems that give perfect results even with low information. The answer to the second question is that these are probably perfectly crafted pre-loaded solutions that we do not consider intelligent, but in theory, it is also possible that they are so intelligent that they can solve the task perfectly even with little information. Existing tests suggest that such pre-loaded solutions are much simpler to implement, so we assume this. The first question about earnings growth from different levels is more difficult to answer and is likely to go beyond the scope of the present research. Here, our primary goal is to detect these knowledge increases and separate them from pre-learned abilities. If we can do this, the next step is to define exactly how to compare these increments.

## 4. Materials and Methods

In the research, we take image recognition neural networks as an example and examine what capabilities they can acquire based on different information. Neural networks are widely used for a huge range of tasks such as object recognition in aerial images [32], heart health monitoring [33], or pedestrian segmentation [34]. Similar to ARC tasks, we perform certain transformations on certain shapes, and understanding and correctly applying these transformations is the knowledge with which the tasks can be solved. Different instances of the neural network are given different teaching sets and test sets, and we examine how successfully they perform on the test set. Both sets contain images on which we have performed certain transformations, so we will examine whether a particular transformation has been understood by the neural network.

Learning to transform can be avoided if the appropriate information is provided in the training set. Take rotation as an example. The system may not understand that the shape can be rotated anywhere but separately notes for each rotated copy that it is a variation of the original shape. Thus, if you encounter the same variations in the test set, you will achieve a good result. Therefore, we consider tests to be convincing where you can apply a transformation that you have not yet seen exactly; for example, you have only seen rotation at a different angle, or you have only seen rotation at another shape.

Neural networks may not be capable of this kind of intelligent behavior, and they may not be able to learn transformations if they have not seen something quite similar. The research focuses on how similar they should see this, that is, how much they can apply the knowledge learned in one task to another, which characterizes their abstraction power. In addition, it should be borne in mind that neural networks have a well-defined and human-made structure. Different layers follow each other, performing each step of information processing. By modifying the individual layers, it is also possible to determine the structure of the neural network capable of recognizing the given transformation. If indeed, the application of a network with a certain structure allows the recognition of a given transformation, it again only raises the question of the pre-coded solution. We also see an example of this among the pre-written solutions, where the neural network was required to have symmetrical layers and thus developed an invariant network for rotation.

### 4.1. Original Shapes

The best-known example of image recognition tasks is the recognition of handwritten digits in the MNIST database, which is also typically solved with neural networks. The MNIST database contains 28 × 28 images. In the present research, we will work with images with a similar structure, which has several advantages. We can use the neural networks tested in the MNIST database and compare our results in the two tests. Furthermore, in the future, it is possible to carry the handwritten digits of the MNIST database through the transformations tested in the research and test how they can be recognized by an intelligent system.

In the MNIST database, the darkness of each field is indicated on a scale of 0–255, and we color the pixels in the range of 0–9. In both cases, the values are scaled to the 0–1 range before being processed by the neural network. By the way, ARC tasks also use 0–9 coloring.

The use of handwritten digits has significant drawbacks, such as the difficulty of recognizing each digit is very different, but more importantly for us, these shapes are invariant for certain transformations. For example, the 8th digit rotates 180° to return itself, and even the 6th digit rotated 180° returns a 9th digit, which would make it completely impossible to study the rotational transformation. Therefore, new shapes are introduced instead of Arabic numerals. The most important aspect of the new shapes is that there are no invariants for rotation, and the rotated copy of any shape must be different from all the original shapes. In addition, it is advantageous if the shapes are approximately equally difficult to recognize, so there are no outstandingly similar shapes that are particularly difficult to distinguish from each other. Finally, it is also important to keep the shapes as simple as possible to facilitate implementation.

**Figure 1:**
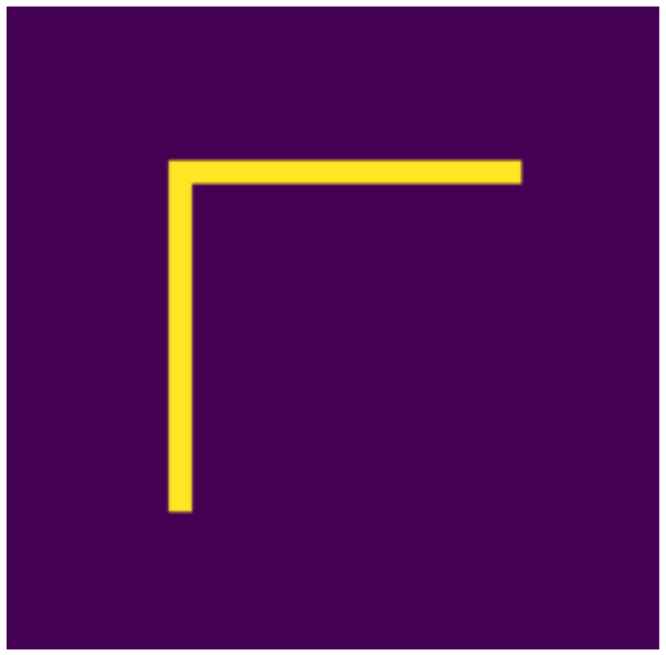
Null.

**Figure 2:**
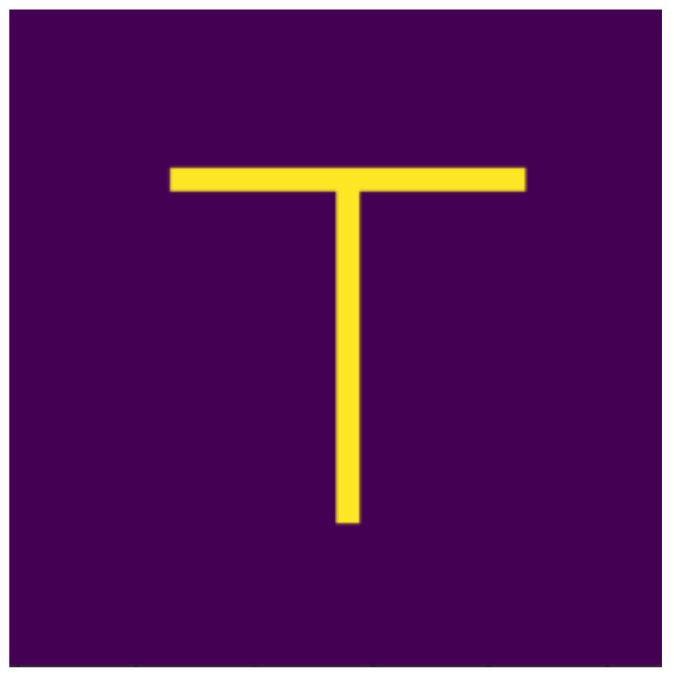
One.

**Figure 3:**
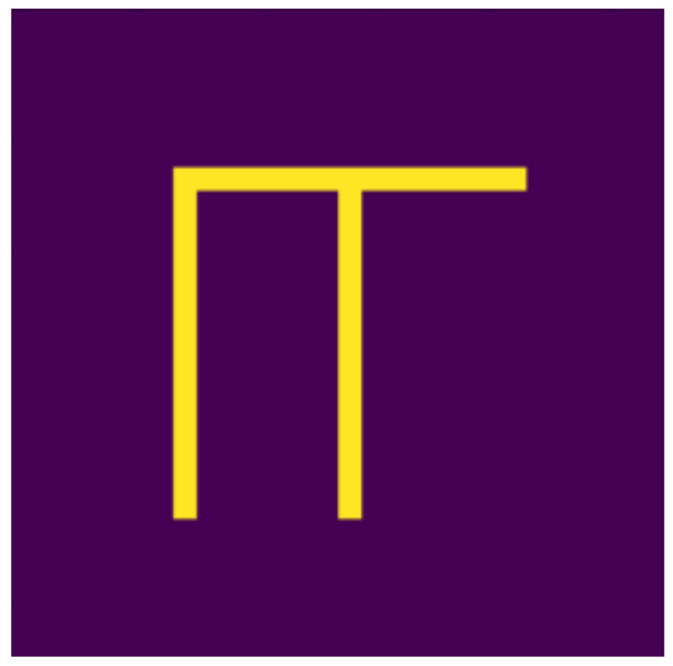
Two.

**Figure 4:**
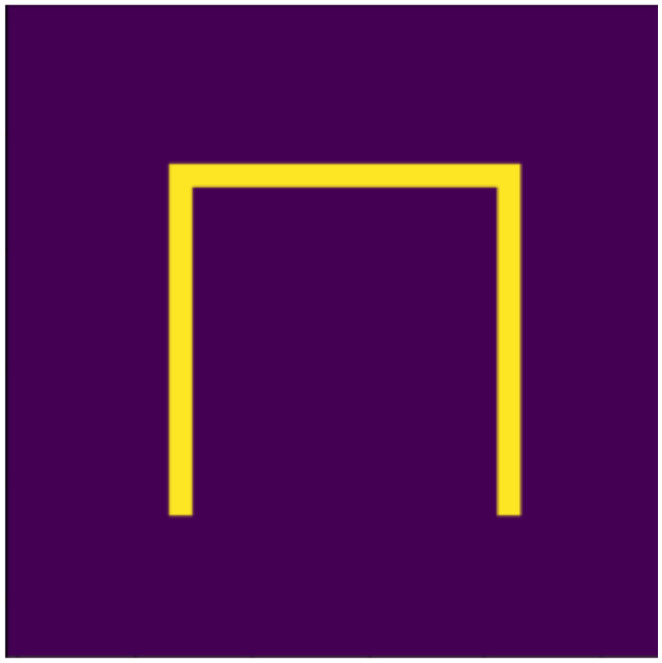
Three.

**Figure 5:**
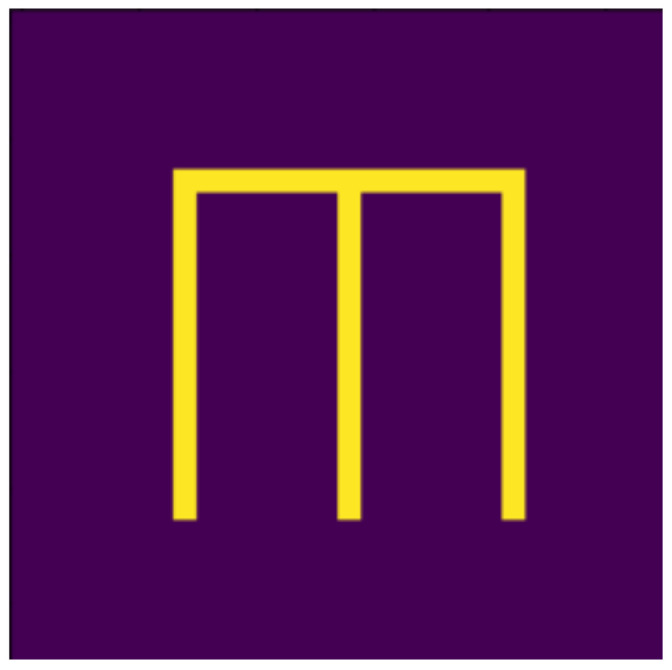
Four.

**Figure 6:**
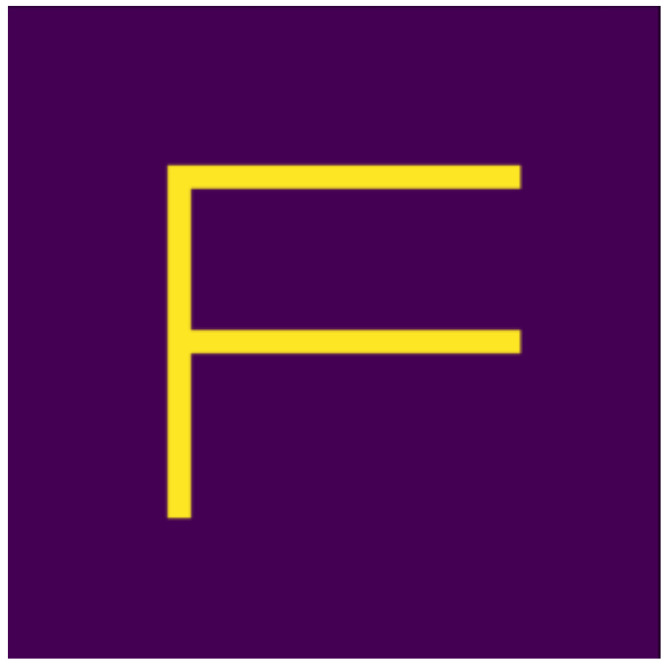
Five.

**Figure 7:**
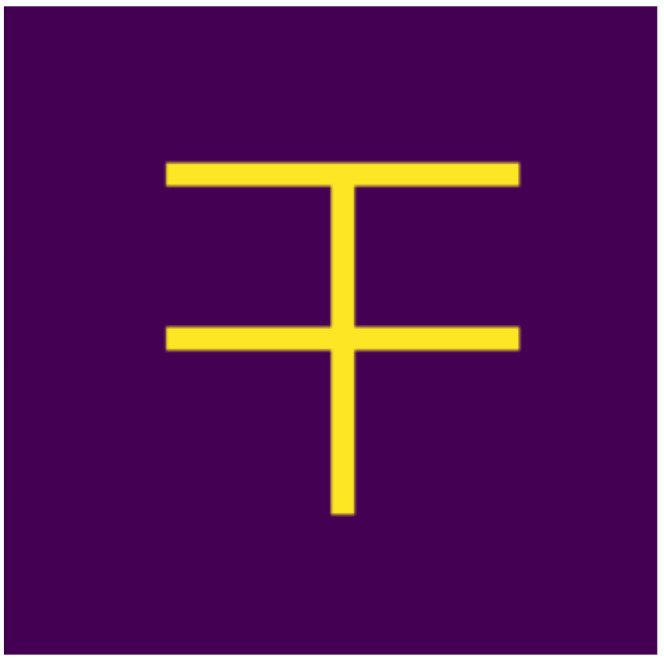
Six.

**Figure 8:**
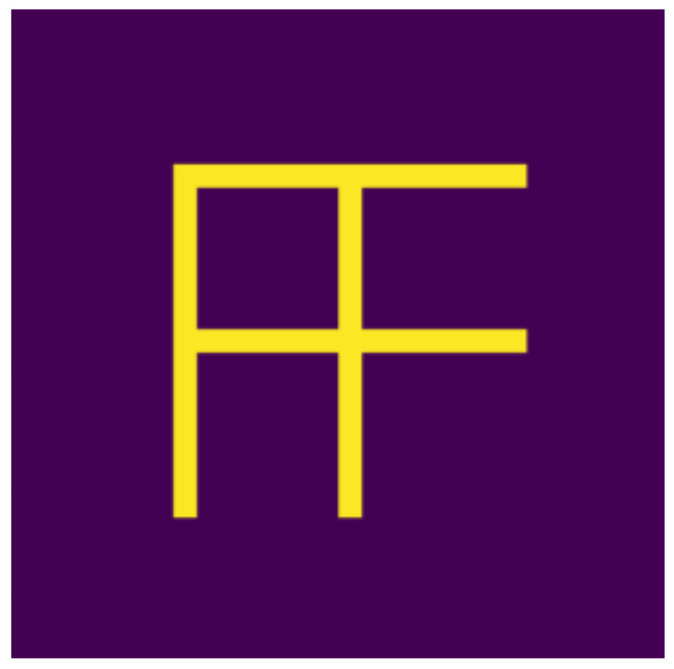
Seven.

**Figure 9:**
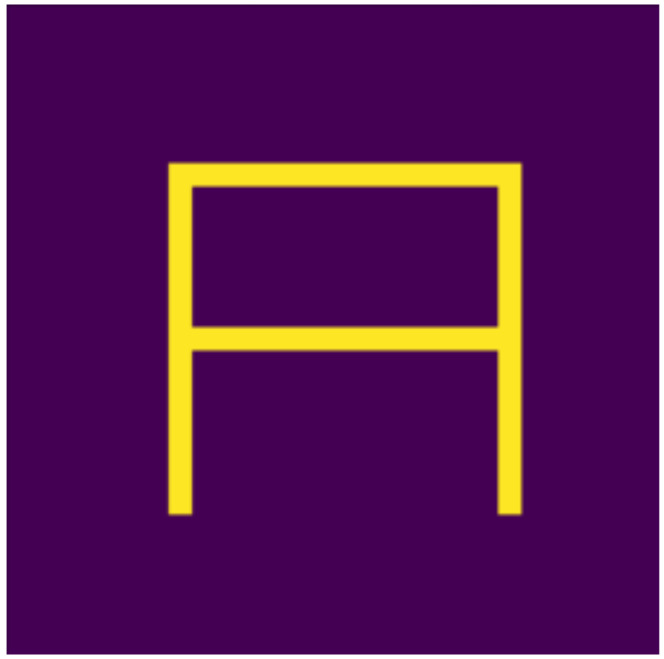
Eight.

**Figure 10:**
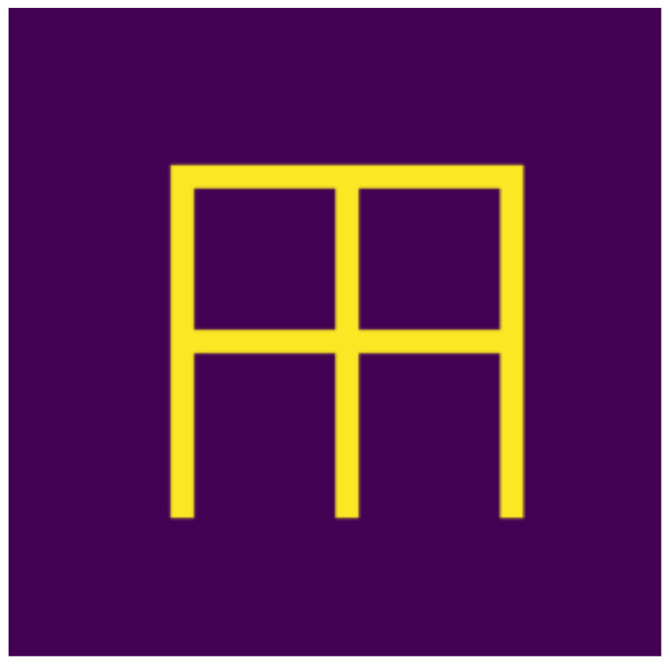
Nine.

Based on the aspects listed above, we defined 10 digits, which can be seen in Figure 1, Figure 2, Figure 3, Figure 4, Figure 5, Figure 6, Figure 7, Figure 8, Figure 9 and Figure 10, using 3 horizontal and 3 vertical lines that are easy to implement and transform yet invariant for rotation, and each can be well distinguished from the other. We will perform various transformations on these shapes to create learning and test sets from the transformed and original shapes.

### 4.2. Examined Transformations

**Figure 11:**
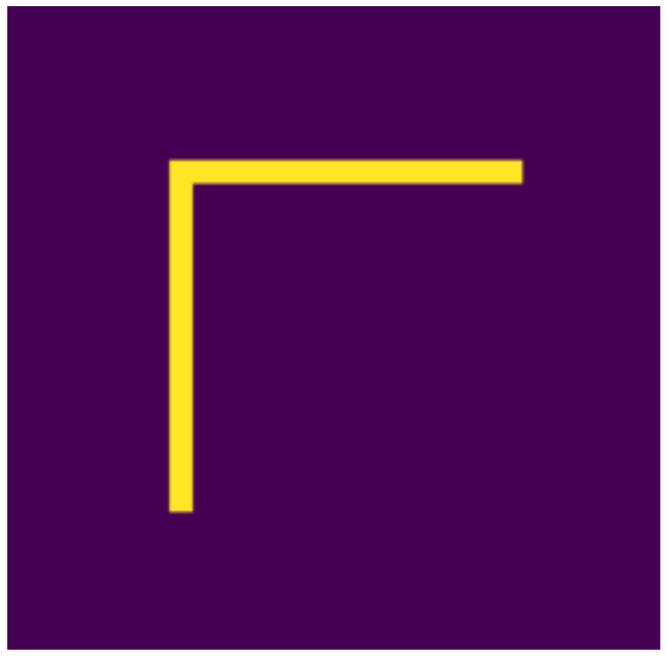
Original.

**Figure 12:**
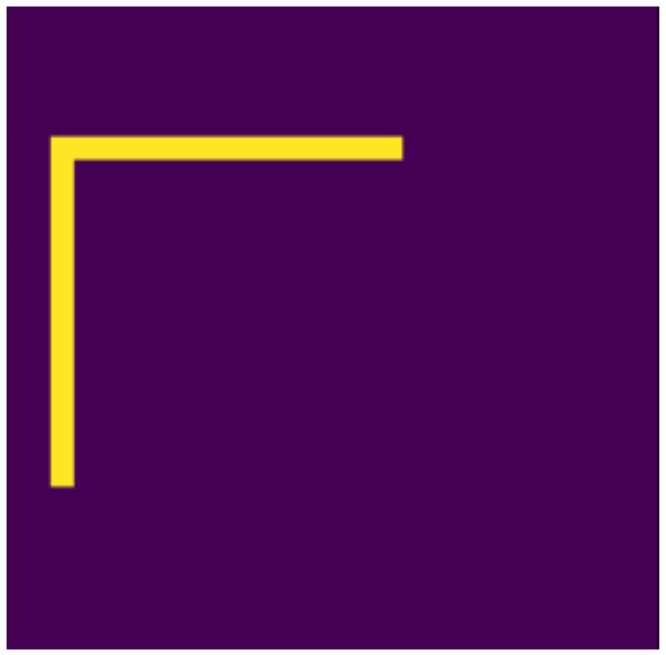
Moved.

**Figure 13:**
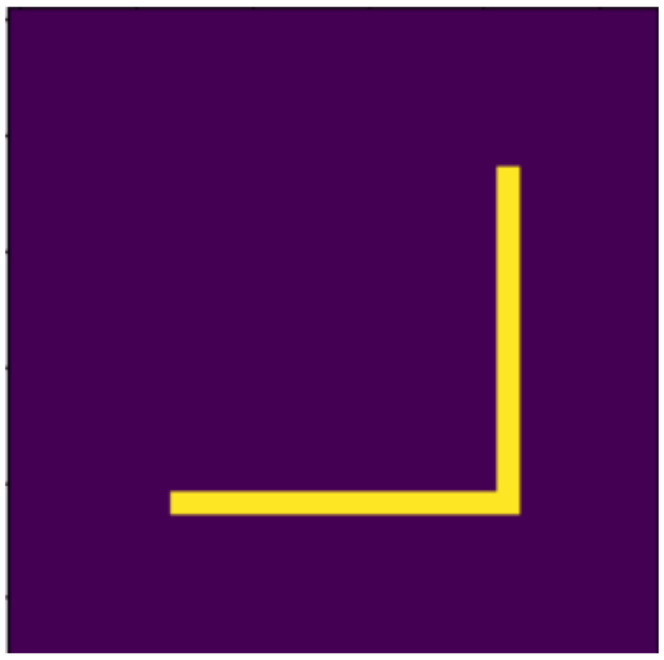
Rotated.

**Figure 14:**
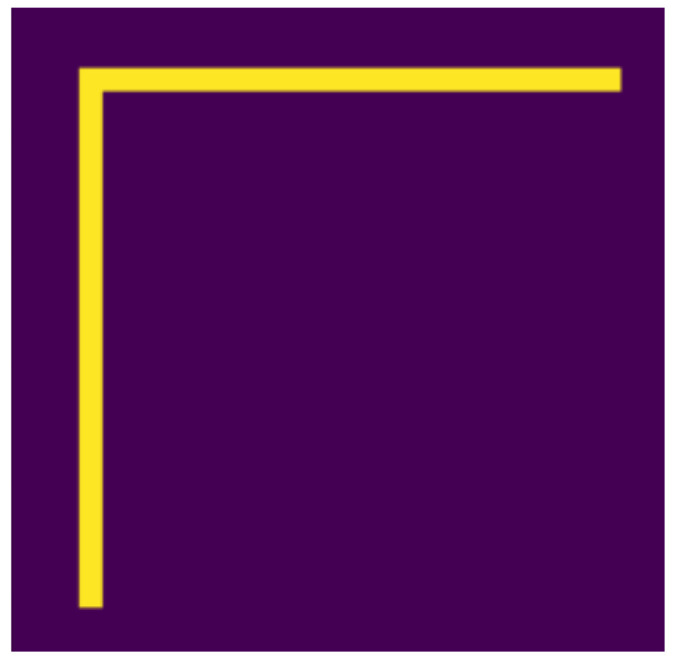
Resized.

**Figure 15:**
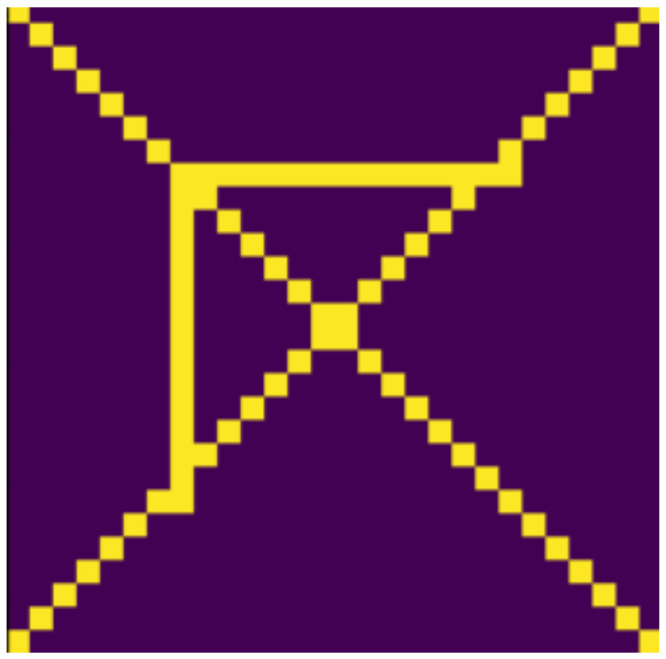
Diagonals added.

**Figure 16:**
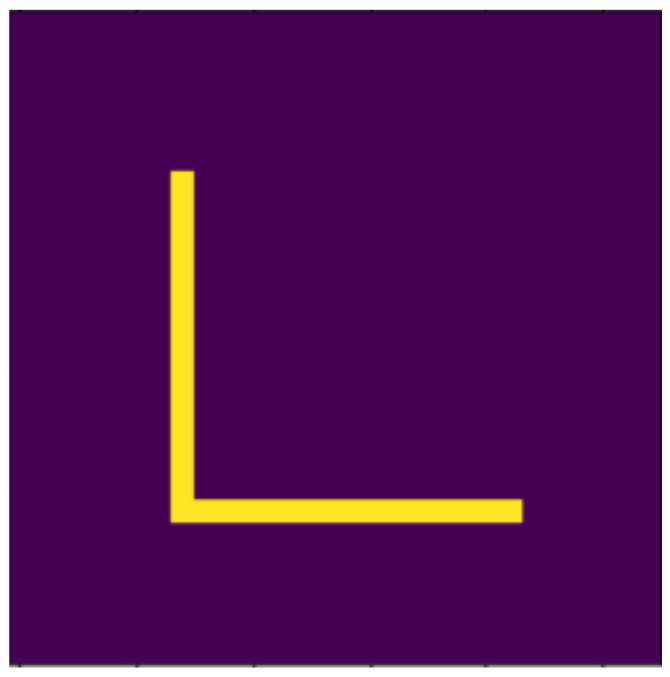
Mirrored.

In the following, we present the transformations we want to perform on an example figure (Figure 11). These are translation (Figure 12), rotation (Figure 13), resizing (Figure 14), add diagonals (Figure 15), and mirroring the image (Figure 16). Adding diagonals means adding the two diagonals to the image, drawing about an X-shape on it. This transformation is called a diagonal for simplicity in the following. In addition to the above, we also use noise as a transformation, but we will not examine it separately because there are already many different solutions for filtering out noise. On the other hand, we expect an increase in the generalization capacity of the neural network from the introduction of noise, and it also helps to make each generated image different from each other, as the number of variations is very limited for other changes, while the number of possibilities is almost endless. Therefore, the emphasis is on how effectively the system can learn the other four transformations, and the introduction of noise can be a tool for this.

**Figure 17:**
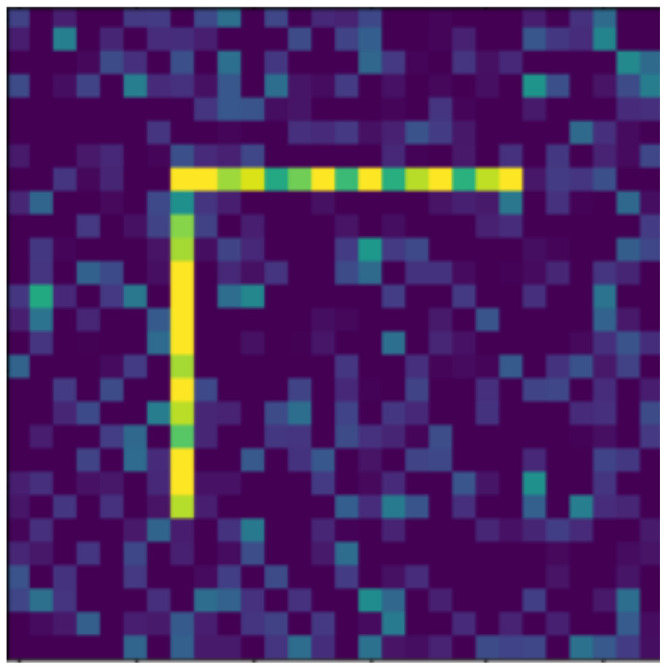
Level 2 noise.

**Figure 18:**
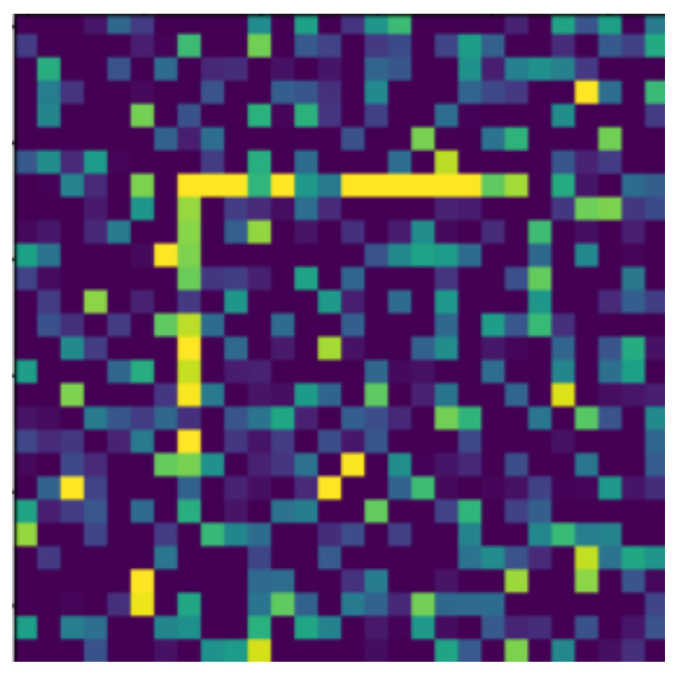
Level 4 noise.

Random Gaussian noise was used as the noise. That is, for each pixel, a random number was generated, and the pixel value was changed with the random number as long as it did not exceed the maximum or minimum value of the pixel. Random numbers generated according to a normal distribution around 0 usually change the pixel value only slightly, but in some outliers, they can change it drastically (see Figure 17). By default, pixels contain only maximum and minimum values, so there is no change in 50% of the cases for a given pixel. The noise can be of different intensities, 2 (Figure 17) or 4 (Figure 18) intensities in our examples, where the intensities are equal to the standard deviation of the random values.

An important difference between the five transformations (rotation, displacement, resizing, adding diagonals, mirroring) is how many different outcomes they possibly have. In the case of rotation, this is 4 since in the square image we can rotate the shape according to the north, east, south, and west orientations. When moving, this number is 196, which is the square of 14, because we can adjust the shape to 14 different positions on both the x and y axes. In the case of resizing, the output can be 14 different sizes, of which 5 are smaller and 8 are larger than the original shape, so the chance of zooming in is slightly higher than that of zooming out. This can be an important factor in recognizing transformations, as the more options you have, the less likely you are to get the original object back, meaning the task will be more difficult. Adding diagonals and mirroring the object is deterministic, and these only have one possible outcome.

### 4.3. Image Recognition System

The experiments were performed with the same predefined neural network. Of course, a different neural network or intelligent system would have produced different results. The structure of the neural network we use is as follows:

- 5 × 5 convolution layer with ReLU (Rectified Linear Unit) activation layer
- 2 × 2 maximum selection;
- 3 × 3 convolution layer with ReLU activation layer;
- 2 × 2 maximum selection;
- Pronunciation regularization of 0.1;
- Matrix array conversion;
- 128 densely coupled neurons with ReLU activation layer;
- 50 densely coupled neurons with ReLU activation layer;
- 10 densely coupled neurons with the Softmax activation layer.

The neural network loss function was defined using categorical cross-entropy, and adaptive momentum estimation (Adam optimization) was used during teaching. This network performs with excellent accuracy of over 99% on the handwritten digits of the MNIST database, where the transformations we implement do not occur, but many random errors occur due to handwriting, and the arc of the digits is always slightly different. Achieving such accuracy on this data set is a huge performance. It took a lot of energy while it was created, and to this day, the MNIST database is used as a benchmark in the field of image recognition software development [35]. However, in this case, similar types of objects appear in the learner and test sets, so the property of interest is not tested here, as local abstraction is sufficient to complete the test.

We briefly describe the function of each layer of the neural network. In the case of a densely coupled layer, all neurons are connected to the previous layer, and their values are the linear combination of the values of the previous layer and the weight matrix of that layer. The convolution layer works similarly, except that each of its neurons uses only a subset of the neurons in the previous layer. For example, in the case of a 5 × 5 convolution layer, one of the neurons arranged in a matrix of the previous layer in a 5 × 5 shape will be the input. The role of the activation layer is essential for neural networks; they break the linearity of the neural network and, thus, ensure that it cannot be traced back to a single layer, which would significantly impair the expressive power of the network. The most commonly used ReLU activation is used. The ReLU function sets the negative values to 0 and leaves the positive values unchanged. The disadvantage is that in the negative range, the gradient will be 0. For the last layer, where only 10 neurons are present, Softmax activation is used. The 10 neurons here can be mapped to each digit, and their value is proportional to the probability calculated from the neural network prediction that the given digit is the solution. Softmax simply selects the highest value, and the neural network will return with that value. In the case of maximum selection, only the maximum value is kept from one area, the other values are omitted. Thus, with a maximum selection of 2 × 2, four neurons determine the value of one neuron in the next layer, which will be the maximum of the values of the previous four neurons. The role of pronunciation regularization is similar to the noise used in images. The value of a certain proportion of neurons is not taken into account (in this case 10%), and the result is determined only based on the other neurons. This, like noise, helps the system prefer the more general solution to the specific one [36].

## 5. Discussion

The measurements were executed in a Python Notebook available in the following GitHub repository: https://github.com/mhudaky/MeasuringAbstraction.git (accessed on 18 August 2021).

Perhaps the first test that comes to mind is how well the neural network can recognize transformed objects in the knowledge of the original objects. The results are included in Table 1. The column headers in the tables always indicate the sample number of the training set. The first line contains the original objects in both the test and training sets. In this case, the network is able to achieve 100% precision, but no abstraction is required to achieve this since the system learns the same images that we use to evaluate its precision.

In addition to the original objects, the system achieved good results even with level 2 noise. Image recognition neural networks can be very effective against noise, and due to their structure, they can be sufficiently robust for a random, not too large variation of pixels. However, when learning on the original objects, this only achieved high efficiency with level 2 noise. Level 2 noise is still very weak (Figure 17), and level 4 noise (Figure 18) is stronger, but the objects are still easily visible to a human observer. As can be seen in the figures, in the case of level 2 noise, it can be determined from virtually every pixel whether or not it belongs to the shape, and the image of the shape is quite clearly drawn. In the case of level 4 noise, it is not clear at the pixel level whether the given frame is part of the shape or not, but looking at the whole image, the shape is clearly drawn. In the case of level 6 noise, it is really difficult to see the shape, but a human test subject, knowing the original shapes, could choose which shape to see the noisy image of. Thus, the neural network can only remain robust with clear noise at the pixel level if no noise is experienced in the teaching set.

For the other transformations, very different results can be observed. We have to take into account that even in the case of accidental guessing, the system achieves a result of 10%, so we can only take into account the performance above. That is, for example, a result of 40% means that the system gave a correct result in 32%, and in the remaining 78%, randomly selected, the answer was found in 10% of cases. Thus, calculated at 32%+78%×0.1, you can achieve a result of about 39.8%.

We teach the network with a relatively low input pattern since the same untransformed objects are repeated in the teaching set, i.e., the system cannot learn much new by increasing the teaching set. However, based on the first line, we also see that for 500 images, it is still only necessary to annotate the original shapes, which means 50 repetitions for the 10 displayed images. This value is highly dependent on the network settings, which in our case, iterates 10 times across the training set.

Both rotation and motion transformations show a characteristic symptom of over-learning, in which case the system performs worse with a larger input sample. Although these decreases are very small, the results are roughly stagnant at around 40% for rotation and around 25% for displacement. In the case of resizing, we do not see a significant increase either, and the result achieved by the neural network creeps up to about 58%. The only transformation that shows a significant increase according to the table is the addition of diagonals. Indeed, the shape itself does not change here, only two diagonal lines are inserted behind it, so it is logical that with longer learning of the original objects, a stricter attachment develops to each line so it can give a higher proportion of correct answers. If the system can ignore the lines that appear next to the shape, that is an important and useful feature, but it does not indicate the intelligent behavior we are looking for. Tested on a larger number of samples, we can see that the neural network we use can achieve maximum efficiency of around 70% even on this transformation if only the original objects are included in the training set.

So according to Table 1, we can state that rotation, displacement, and resizing are not transformations that would be pre-encoded in the network; if they cannot be known from the set of learners, they cannot be known effectively. recognize. However, some similarities may occur between the original and the transformed objects, so when examining whether a system can learn a transformation based on a set of learners, we must consider that these similarities can be used to identify objects without understanding the transformation. Thus, it is useful to estimate the proportion of these hits without real learning and to use them as a benchmark, for which Table 1 is suitable.

In Table 2, we tested the results that can be achieved if we teach the system on transformed objects and need to recognize similar transformed objects in the test set. Since there is nothing to prevent the same shapes from being included in the learner and test sets, it is not surprising that with the right amount of data, the system can achieve nearly 100% results. With a higher amount of images, the system is more likely to encounter the image in the test during learning, so of course, as the set is increased, the accuracy increases significantly. However, we also see that in the case of different transformations, the values when close to 100% are reached significantly different. This is due to the fact that transformations can have different numbers of variations. Indenting diagonals is deterministic, we only have one option, so this can be learned very quickly for the neural network. There are only four variations of the rotation, so this can also be mastered relatively quickly. There are 14 options for resizing and 196 for moving, so these are learned more slowly by the system. The data in Table 2 correlate well with how many variations of a given transformation are possible. However, here too, we have to note, in the case of the previous table, that abstraction was still not needed, as the web only recognized the shapes already seen in the teaching set.

In the case of Table 3, we made the learning algorithm more difficult by assigning level 4 noise to the images of both the training set and the test set. This means that the system cannot learn on exactly the same images that will appear in the test set, but it can on very similar images. Many different outputs can occur after noise, but these outputs differ only slightly, whether they are relatively easily distinguished by a human observer or by an intelligent system. However, these results show a significant reduction in efficiency for a similar number of samples, i.e., the combined application of the transformation to be learned, and the noise really makes the task more difficult. However, it can also be assumed that due to the application of noise and thus the exclusion of cases that are too clear, the system is forced to find a more general solution, which would not be necessary in the case where the images were identical.

The data reported in Table 3 were tested on transformed and noisy objects. Examples of this are shown in Figures 20–24, with the rotated, moved, resized, and diagonal objects, respectively. In this case, level 4 noise was used in addition to the transformations. In such cases, the transformation is always applied first, and the noise is applied to the resulting shape. As you can see in the pictures, at level 4 noise, the outlines of the shape can still be clearly seen, but some pixels may already be blurred.

### 5.1. Transformation Analysis

In the next two chapters, we examine the extent to which the neural network tested can apply the transformations seen on each shape to other shapes. To do this, we will provide different amounts of the transformed form of the shapes in the training set. Namely, we will test with 0, 2, 5, 8, and 10 level transformations, which means that the first 0, 2, 5, 8, and 10 shapes are transformed in the training set, but the test sets are similar in all cases. Here, in all cases, all 10 shapes will be transformed. The addition of noise is expected to increase the generalizing ability of the neural network.

However, for the data in the tables, it is extremely important to consider the proportion of untransformed shapes in the test sets. This is because some of the transformed shapes will be the same as the original shape since transformations have a finite number of variations, and the finite number of variations includes the original shape. Since the chances of each variation are equal, it is assumed that the original shape is returned after the transformation is performed is proportional to the reciprocal of the variations. Thus, if we want to get how much of the shapes in the test set are known by the neural network from the training set, we take the ratio of the originally transformed objects and add the quotient of the remainder and the number of variations. The number of variations in moving and resizing is large, so here, this effect will be negligible. In the case of rotation, four types of variation are possible, so a quarter of the transformed objects are the same as the original object. This is a very significant effect, which we will also see in the results of the tables. In the case of diagonalization, only one variation is possible, but here, this variation does not correspond to the original shape, as there was no diagonal behind the original shape. Therefore, this effect does not need to be taken into account at all when inserting diagonals.

Another important effect is the random hit rate. Since there are only 10 different shapes, if we accidentally choose one of them, we still have a 10% chance to hit the right shape. This effect occurs equally for all transformations.

Therefore, we need to consider the previous two effects and only want to attribute the intelligent behavior of the neural network that could not be achieved with either the untransformed shapes in the test set or the random hits.

### 5.2. Transformation Analysis on Level 2 Noise

**Figure 19:**
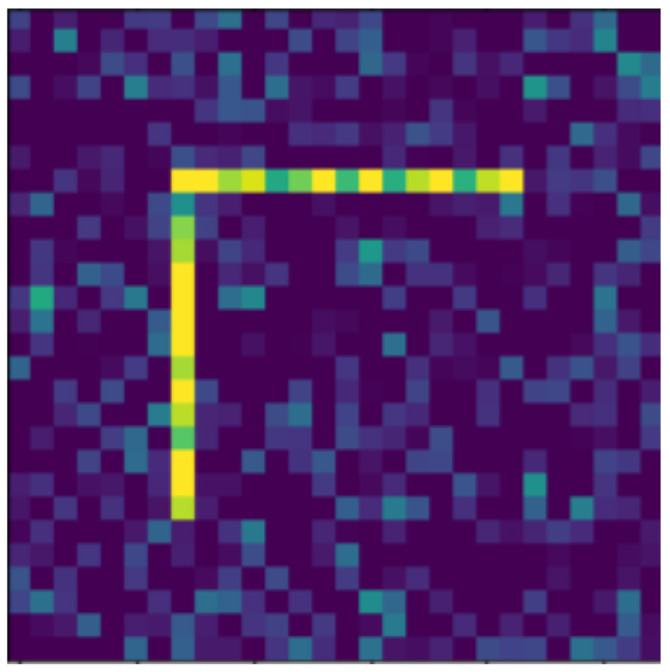
Original object, with level 2 noise.

**Figure 20:**
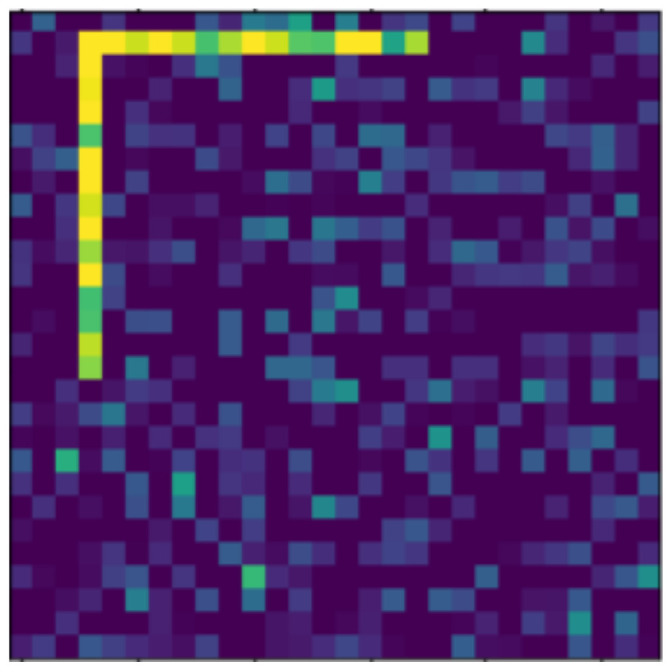
Moved object, with level 2 noise.

**Figure 21:**
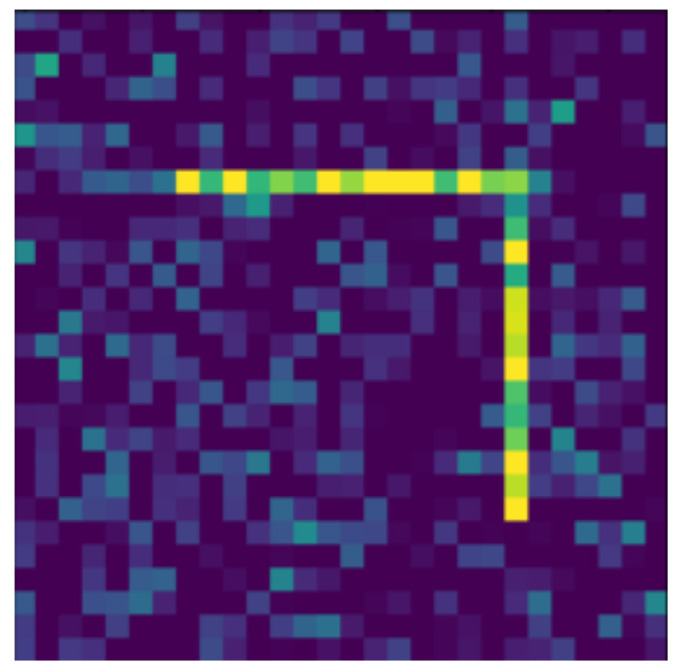
Rotated object, with level 2 noise.

**Figure 22:**
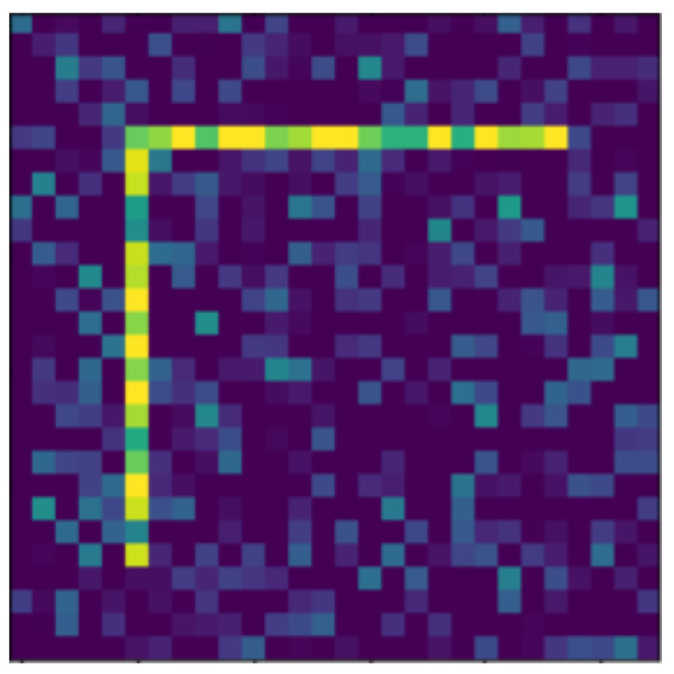
Resized object, with level 2 noise.

**Figure 23:**
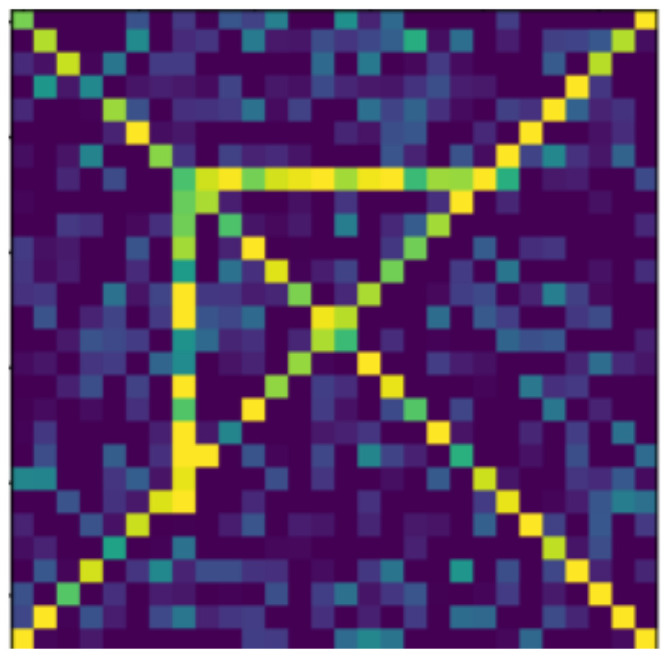
Object with diagonals added, with level 2 noise.

**Figure 24:**
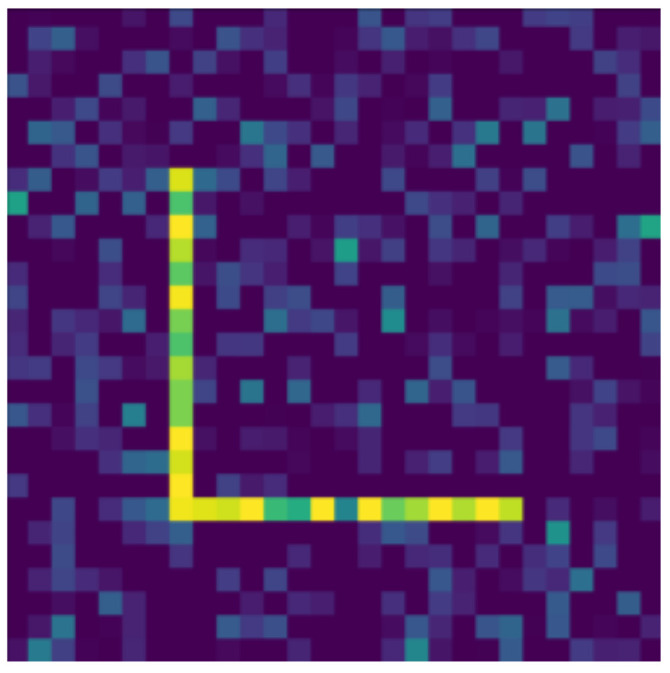
Mirrored object, with level 2 noise.

Tables 9–13 were recorded with level 4 noise. Figure 19, Figure 20, Figure 21, Figure 22, Figure 23 and Figure 24 show how the transformed objects look with level 2 noise.

Thus, in the case of Table 4, we passed all the objects untransformed to the training set, but the shapes in the test set were, of course, transformed. Based on an earlier derivation, in the case of the rotation transformation, a quarter of the shapes of the test set are the same as the original shape and thus are included in the training set. Thus, we can easily achieve a result of 25%, and we can randomly guess 10% of the remaining 75%, i.e., we can achieve 32.5% without inferring the correct answer to unknown shapes. Based on a similar derivation, we can find 16.4% for the movement and 10.5% for the resizing without understanding the transformation. Fortunately, we are able to achieve slightly more than this based on the table, so for transformed shapes, the network shows some preference for the already-known untransformed version of the same shape compared to a completely different shape. Of course, this does not mean that you have understood the transformation; the transformed objects are a little more similar to the original object than a completely different one, and neural networks are extremely effective at filtering out small similarities as well.

According to Table 4, we measured the lowest result on the shifted objects, the medium on the rotated and resized shapes, and the best on the diagonals. Applying the corrections described earlier, it was about 7% for the moved, 14% for the rotated, 38% for the resized, and 61% for the diagonaled, which was actually recognized by the network. The fact that the moved are the hardest to recognize and the diagonal the easiest is consistent with our assumptions so far. When moved, all the lateral lines of the shape are moved to another location, and the number of variations of the transformation is also the largest here. At the diagonal, each page stays in its usual place, and the number of variations is only one. The data in the table also correlate well with the previously measured data. In the case of the mirrored objects, we recorded good results, but at a higher sample amount, the network was overfitting, and its results started to decrease.

Comparing the data in Table 5, we are looking for improvements of at least 20% compared to Table 4. Here, we transformed 2 out of 10 shapes into the training set by transforming them. Thus, the web can recognize 20% more without learning, but at the same time, the significance of the two effects mentioned earlier (transformed objects present in their original form and random hits) decreases. Therefore, the expected increase will ultimately be less than 20%, provided that the network has not been learned to apply the transformation. However, an increase above 20% indicates that the system also achieved higher results on shapes that did not see their transformed form, as the upper limit of the effect of the improvement on the shapes shown in the transformed form is 20%.

However, the data in Table 5 show quite different results, and there are no improvement predictions of the neural network. Moreover, according to the data in the table, the accuracy of the predictions decreased. This is possible because the shifted image of one shape may be more similar to the shifted image of another shape than to its original version if some lines of the two shifted shapes coincide. Thus, instead of learning the logic of transformations, the neural network categorized similar figures at the pixel level into one category. Our former assumption is also supported by the fact that the predictions stagnated in the case of rotation, where the lines of all shapes are centered in the middle of the whole image. In the case of the mirrored objects and the ones with diagonals added, the results were good without transformed objects in the training set. Adding transformed objects confused the network and resulted in a significantly worse outcome.

For Table 6, we want to see an improvement of at least 30% over Table 5 so that we can say with confidence that performing transformations on certain shapes have increased the hit rate on other shapes as well, which we almost reach in the case of shifted objects.

As in the previous case, we want to see a 30% improvement between Table 6 and Table 7. This 30% threshold could only be reached for mirrored objects and the objects with diagonals added. It is necessary to add that in the case of objects with diagonals added, the uncertainty of the values is very high, but the highest value of Table 6 is 57.20%, while in the case of Table 7, this number is 97.00, which indicates that neural network performance has also improved on shapes about which it did not receive extra information during learning. From the nearly 100% result measured in the second case, it can be seen that the neural network had learned this transformation almost perfectly and was able to apply it well to each shape. Here, the training set contained the transformed version for eight shapes, so there were two shapes for which the network could correctly filter out the transformation without seeing it on that shape before. That is, learning on eight shapes, the network was able to generalize the rule that inserting diagonals does not change the shape type and applied it to the other two shapes so that the same did not happen for five transformed shapes, i.e., the correct prediction cannot be the result of a pre-written solution or other non-intelligent mechanisms. This is indeed a task that requires abstraction, even if it has only been achieved in a very special situation with the neural network. In the case of the mirrored objects, the uncertainties are low, and the improvement in the results is clearly higher than 30%. The Increment is from 50% to 85%, which is less significant than in the case of objects with diagonals added, but acquiring some knowledge was still a necessity to reach these results.

The only thing that can be seen from Table 8 is that the network can learn all the transformations perfectly if it can learn it in all 10 shapes through the training set. This is the expected behavior for neural networks; in fact, this table only proves that our neural network works well, and it can meet the properties expected of it.

### 5.3. Transformation Analysis with Four-Level Noise

Table 9, Table 10, Table 11, Table 12 and Table 13 were recorded with level 4 noise. Figure 20, Figure 21, Figure 22 and Figure 23 show how the transformed objects look with level 4 noise.

Due to the higher level of noise, the neural network is looking for a more general solution and thus can give more accurate sermons. However, higher levels of randomly generated noise can blur some images so much that they cannot be recognized by the network. In Table 2, we can see that we experience a negligible prediction deterioration at level 2 and level 4 noise, but this will be significant at level 6 noise. Therefore, we no longer ran the transformation analysis with six levels of noise, as we can expect significantly worse results in this case.

We are looking for data where we obtained better predictions with level 4 noise than with level 2 noise, as this proves that we have indeed succeeded in increasing the generalizability of the model as a result of increasing the noise.

Comparing Table 4 and Table 9, we can see that while the accuracy of the prediction was indeed increased in the case of rotation drawing diagonals and mirroring, the accuracy of the displacement and resizing decreased. This also shows that there are two opposite effects with increasing noise: On the one hand, it can increase the generality of the solution that is predominant in rotation and diagonals, and on the other hand, due to the randomness of noise, some images can be so blurred that they can be difficult to recognize. In the case of displacement and resizing, this second effect was decisive. It is also interesting to examine how the accuracy of each transform changes in Table 9 as a function of the sample numbers. When diagonals are inserted, we give better predictions at a low number of samples even at a lower noise level, which seems logical, as the number of samples was not large enough for the network to successfully learn to filter out even higher levels of noise. However, it is surprising that in the case of the rotation, this effect does not occur and at level 4 noise we obtain stable, almost identical values for 1000, 2000, 5000, and 10,000 samples.

Comparing Table 5 with Table 10, we similarly see that a slight improvement in rotation and a stronger improvement in mirroring and adding diagonals followed an increase in noise level. In the case of movement and rotation, the values did not change significantly. Comparing Table 4 and Table 10, the decline in the transformation of the first two shapes was milder than in the case of level 2 noise, and in the case of the rotation and displacement, the predictions even improved.

Table 6 and Table 11 show very similar results, in the case of rotation and resizing, weaker noise was preferred. At the other three transformations, the stronger noise resulted in better performance. The trends are similar to those observed at level 2 noise. In the case of the mirroring and adding diagonals, the predictions become weaker with the increment of transformed objects in the training set, but at other transformations, the prediction improved.

For a level 8 transformation, level 4 noise (Table 12) performed slightly weaker than level 2 (Table 7) in the case of displacement and resizing but stronger in the case of mirroring and adding diagonals. Compared to the lower transformation, the accuracy of each transformation increased significantly, and the special case that was already observed in the case of level 2 noise also appears here. In the case of a level 5 transformation, the highest result for the insertion of diagonals was 55.6%, while in the case of a level 8 transformation, it was 98%. From this, we can see that the accuracy of the prediction has improved by more than 30%, which is higher than the proportion of changed objects, thus proving that the network was able to understand the transformation at some level and apply it to other objects. Similarly, in the case of mirroring, we can see a smaller improvement with less uncertainty. Furthermore, this gap is more significant at the increased noise, reaching an improvement of 40%.

In the case of a level 10 transformation, we also see here that the network was able to learn the transformations roughly, but an error of 1.0–3.4% occurs due to stronger noise.

## 6. Conclusions and Future Works

Our goal was to create a system in which we can effectively measure the abstraction capability of different software. Previous tests of a similar nature have been based on how much knowledge the software has been able to acquire in a given environment. A problem with this approach is that learning can be avoided if the programmer pre-encapsulates the knowledge into the software. This is easy to do if the programmer is familiar with the test interface, so they usually try to keep the test cases secret. Our approach avoids this problem by testing the behavior of software in several different environments, allowing us to filter out pre-written solutions.

We can test the intelligence of image recognition software by making transformations on the shapes shown in the images, and understanding and correctly applying these transformations is the knowledge that the software must acquire. If the software can apply the transformation correctly without seeing it in the training set, then it is assumed to be a pre-written solution. We think that the software understood the transformation if it started to apply it correctly as a result of increasing the proportion of transformed objects in the training set. This also allows us to test the conditions under which the software can understand each transformation. The system needs to be able to perform the transformation on an object on which it has not yet seen that transformation; otherwise, it would be sufficient to memorize the transformed shape separately in order to perform the task.

To test this, we examined several different cases in the Discussion section. We registered two very specific occasions where the transformation was understood and recognized in a new environment. Mirroring eight out of ten shapes or adding diagonals to eight out of ten made the network learn these transformations and apply them correctly to the remaining two shapes as well. Based on this, we can state that the neural network we tested is incapable of understanding abstract transformations such as rotating, moving, or resizing a shape. Therefore, if such transformations are used in a different environment than usual, it will not be able to recognize the transformed shapes.

The method detailed in the paper can be used to test any other image recognition software that can be taught on a training set and tested on a test set. The technique is based on showing different forms of transformations in the teaching set and testing on the test set whether the given teaching set was sufficient to understand the transformation.

The advantage of this method over similar methods so far is that we can filter out solutions written in advance by the programmer and accept only those solutions that have been independently developed by the image recognition software.

The method described in the paper can be further developed in many ways.

The same properties should be examined with another image recognition software, which may be a neural network with a different structure or completely different software. Drawing on the lessons of the present paper, it is possible to solve this in a more transparent by removing the original objects from the possibilities when applying transformations. This is because the presence of original objects distorts the results, and the proportion of original objects that were easy to recognize must be recalculated.

A very interesting application of the method is that by changing the image recognition software, we want to map which component of the software allows us to recognize certain transformations. So, for example, in the case of the solutions mentioned in the Hard-coded solutions chapter, we can test what pre-written solution we can use to recognize transformations. This is different from what we have done so far, as we did not specifically look for pre-written solutions; we tried to filter them out and ignore them. Similarly, if we find software that can learn transformation as described, which demonstrates its abstraction capabilities, we can test different software to test what structure of the software can provide this behavior.

## Figures and Tables

**Table 1:** Training set: Original objects; Test set: Transformed objects.

	250	500	1000	2000
Original	91.60 ± 12.08%	100.00 ± 0.00%	100.00 ± 0.00%	100.00 ± 0.00%
Rotated	40.40 ± 8.36%	42.80 ± 3.87%	40.00 ± 3.35%	36.00 ± 3.29%
Moved	24.00 ± 3.16%	26.40 ± 2.33%	25.40 ± 1.62%	25.80 ± 1.60%
Resized	44.20 ± 7.96%	56.80 ± 4.96%	56.60 ± 5.85%	58.80 ± 2.48%
With diagonals	36.00 ± 10.20%	38.00 ± 7.48%	47.60 ± 18.94%	55.40 ± 19.17%
Mirrored	47.60 ± 19.97%	59.40 ± 12.18%	69.80 ± 17.67%	51.60 ± 19.02%
Level 2 noise	74.60 ± 13.28%	96.80 ± 2.56%	94.80 ± 5.19%	99.00 ± 0.00%
Level 4 noise	31.00 ± 13.43%	22.20 ± 2.14%	27.80 ± 5.19%	28.40 ± 3.56%
Level 6 noise	14.80 ± 4.17%	12.40 ± 1.50%	16.80 ± 0.40%	16.80 ± 0.75%

**Table 2:** Training set: Transformed objects; Test set: Transformed objects.

	250	500	1000	2000
Original	93.80 ± 8.11%	100.00 ± 0.00%	100.00 ± 0.00%	100.00 ± 0.00%
Rotated	45.20 ± 2.79%	76.00 ± 3.58%	100.00 ± 0.00%	100.00 ± 0.00%
Moved	26.20 ± 4.92%	38.00 ± 3.52%	78.80 ± 6.52%	98.00 ± 0.63%
Resized	66.60 ± 10.52%	87.80 ± 2.40%	97.20 ± 0.75%	99.60 ± 0.49%
With diagonals	77.40 ± 7.61%	97.80 ± 4.40%	100.00 ± 0.00%	100.00 ± 0.00%
Mirrored	95.60 ± 5.39%	100.00 ± 0.00%	100.00 ± 0.00%	100.00 ± 0.00%
Level 2 noise	78.20 ± 7.60%	99.80 ± 0.40%	100.00 ± 0.00%	100.00 ± 0.00%
Level 4 noise	53.40 ± 11.04%	79.60 ± 8.31%	97.60 ± 1.85%	99.00 ± 0.00%
Level 6 noise	22.40 ± 9.09%	47.80 ± 9.52%	74.40 ± 2.87%	90.80 ± 0.98%

**Table 3:** Training set: Transformed objects with level 4 noise; Test set: Transformed objects with level 4 noise.

	250	500	1000	2000
Original	52.00 ± 3.74%	82.40 ± 7.00%	98.40 ± 0.80%	98.80 ± 0.40%
Rotated	22.00 ± 1.10%	36.00 ± 5.02%	54.60 ± 4.63%	79.80 ± 3.76%
Moved	12.00 ± 2.45%	17.20 ± 1.33%	26.60 ± 4.63%	56.60 ± 4.88%
Resized	25.40 ± 4.32%	51.80 ± 7.47%	66.20 ± 5.71%	87.20 ± 2.48%
With diagonals	42.00 ± 10.22%	63.40 ± 10.67%	92.20 ± 3.76%	98.80 ± 0.40%
Mirrored	44.60 ± 4.59%	71.80 ± 4.53%	88.00 ± 6.03%	96.60 ± 4.32%

**Table 4:** The number of transformed objects: 0; Level of noise: 2.

	1000	2000	5000	10,000
Rotated	42.80 ± 3.54%	46.80 ± 1.17%	46.60 ± 1.02%	46.80 ± 2.04%
Moved	26.80 ± 0.75%	25.20 ± 1.60%	25.20 ± 0.75%	25.20 ± 1.83%
Resized	53.20 ± 2.56%	53.20 ± 3.06%	52.80 ± 2.56%	55.20 ± 3.43%
With diagonals	88.20 ± 7.30%	87.80 ± 8.08%	92.60 ± 4.80%	93.80 ± 5.38%
Mirrored	90.00 ± 7.69%	83.00 ± 9.36%	93.80 ± 5.42%	84.40 ± 10.25%

**Table 5:** The number of transformed objects: 2; Level of noise: 2.

	1000	2000	5000	10,000
Rotated	42.20 ± 1.94%	43.80 ± 1.17%	48.20 ± 3.71%	46.40 ± 1.85%
Moved	17.80 ± 0.75%	21.60 ± 0.80%	22.80 ± 0.75%	22.80 ± 0.40%
Resized	49.40 ± 3.44%	48.40 ± 3.38%	48.60 ± 5.75%	48.80 ± 6.52%
With diagonals	59.20 ± 9.33%	54.60 ± 9.29%	64.00 ± 5.76%	71.40 ± 9.16%
Mirrored	49.20 ± 16.07%	60.40 ± 9.60%	67.20 ± 12.02%	68.40 ± 8.26%

**Table 6:** The number of transformed objects: 5; Level of noise: 2.

	1000	2000	5000	10,000
Rotated	59.60 ± 4.03%	62.00 ± 0.63%	64.80 ± 2.40%	64.20 ± 0.75%
Moved	29.80 ± 1.17%	46.60 ± 1.02%	50.40 ± 0.49%	51.00 ± 0.00%
Resized	61.00 ± 2.00%	67.40 ± 2.65%	71.00 ± 3.41%	74.60 ± 1.96%
With diagonals	50.40 ± 0.80%	50.20 ± 0.40%	57.20 ± 9.11%	51.40 ± 2.33%
Mirrored	50.00 ± 0.00%	50.00 ± 0.00%	50.00 ± 0.00%	50.00 ± 0.00%

**Table 7:** The number of transformed objects: 8; Level of noise: 2.

	1000	2000	5000	10,000
Rotated	74.00 ± 3.58%	85.20 ± 0.75%	85.60 ± 0.49%	86.60 ± 1.02%
Moved	38.40 ± 4.96%	74.40 ± 2.06%	80.20 ± 0.40%	80.60 ± 0.49%
Resized	77.40 ± 1.74%	82.80 ± 1.47%	85.80 ± 1.17%	88.60 ± 1.36%
With diagonals	81.60 ± 1.74%	86.60 ± 5.61%	92.40 ± 5.92%	97.00 ± 2.61%
Mirrored	80.80 ± 0.98%	82.20 ± 4.40%	82.80 ± 4.66%	85.40 ± 3.93%

**Table 8:** The number of transformed objects: 10; Level of noise: 2.

	1000	2000	5000	10,000
Rotated	83.20 ± 4.07%	99.40 ± 0.49%	99.80 ± 0.40%	99.80 ± 0.40%
Moved	57.60 ± 8.11%	93.00 ± 1.10%	99.00 ± 0.00%	99.20 ± 0.40%
Resized	92.20 ± 2.79%	98.00 ± 0.00%	99.00 ± 0.00%	99.00 ± 0.00%
With diagonals	100.00 ± 0.00%	100.00 ± 0.00%	100.00 ± 0.00%	100.00 ± 0.00%
Mirrored	100.00 ± 0.00%	100.00 ± 0.00%	100.00 ± 0.00%	99.80 ± 0.40%

**Table 9:** The number of transformed objects: 0; Level of noise: 4.

	1000	2000	5000	10,000
Rotated	48.20 ± 1.17%	48.80 ± 1.33%	48.40 ± 0.49%	48.60 ± 0.49%
Moved	20.60 ± 1.62%	20.60 ± 1.74%	19.40 ± 1.20%	19.80 ± 1.17%
Resized	49.00 ± 2.68%	48.80 ± 3.06%	46.00 ± 4.60%	50.40 ± 1.62%
With diagonals	82.40 ± 8.89%	95.60 ± 1.85%	98.00 ± 0.00%	98.80 ± 0.40%
Mirrored	89.20 ± 3.19%	98.40 ± 0.80%	98.80 ± 0.40%	98.80 ± 0.40%

**Table 10:** The number of transformed objects: 2; Level of noise: 4.

	1000	2000	5000	10,000
Rotated	39.20 ± 4.31%	43.60 ± 1.62%	49.40 ± 2.06%	52.60 ± 0.80%
Moved	16.20 ± 0.75%	18.00 ± 0.89%	20.40 ± 0.49%	22.60 ± 0.80%
Resized	37.00 ± 2.97%	44.40 ± 3.61%	47.00 ± 6.07%	48.20 ± 3.31%
With diagonals	56.00 ± 11.52%	79.20 ± 6.24%	89.80 ± 3.54%	88.00 ± 8.63%
Mirrored	42.60 ± 5.78%	60.00 ± 16.53%	66.00 ± 14.39%	87.00 ± 6.75%

**Table 11:** The number of transformed objects: 5; Level of noise: 4.

	1000	2000	5000	10,000
Rotated	43.60 ± 2.42%	58.60 ± 1.02%	65.40 ± 1.74%	63.40 ± 1.02%
Moved	20.40 ± 1.20%	30.40 ± 0.80%	44.80 ± 0.98%	49.20 ± 0.75%
Resized	47.80 ± 3.60%	58.80 ± 2.64%	69.40 ± 4.22%	72.20 ± 2.14%
With diagonals	53.20 ± 2.40%	51.40 ± 1.85%	55.60 ± 5.75%	55.60 ± 7.00%
Mirrored	51.20 ± 2.04%	50.00 ± 0.00%	50.60 ± 0.80%	51.60 ± 2.33%

**Table 12:** The number of transformed objects: 8; Level of noise: 4.

	1000	2000	5000	10,000
Rotated	51.60 ± 3.98%	69.40 ± 2.65%	85.40 ± 1.36%	86.00 ± 1.10%
Moved	21.00 ± 1.55%	40.80 ± 0.75%	70.60 ± 2.06%	77.80 ± 1.17%
Resized	59.80 ± 3.60%	73.80 ± 2.48%	81.20 ± 0.98%	84.00 ± 1.10%
With diagonals	79.40 ± 4.67%	91.20 ± 4.66%	95.60 ± 3.14%	98.00 ± 0.63%
Mirrored	78.40 ± 0.49%	81.80 ± 2.93%	86.60 ± 5.08%	90.60 ± 5.95%

**Table 13:** The number of transformed objects: 10; Level of noise: 4.

	1000	2000	5000	10,000
Rotated	54.00 ± 5.51%	75.40 ± 2.06%	98.00 ± 0.00%	99.00 ± 0.00%
Moved	24.00 ± 4.34%	49.60 ± 8.82%	89.20 ± 1.72%	96.60 ± 0.49%
Resized	69.80 ± 4.26%	88.40 ± 1.85%	95.80 ± 0.75%	97.00 ± 0.00%
With diagonals	93.20 ± 0.40%	99.00 ± 0.00%	99.00 ± 0.00%	99.00 ± 0.00%
Mirrored	97.40 ± 1.74%	99.00 ± 0.00%	99.00 ± 0.00%	99.00 ± 0.00%

## Data Availability

The measurements were executed in a Python Notebook available in the following GitHub repository: https://github.com/mhudaky/MeasuringAbstraction.git (accessed on 18 August 2021).
